# Avian leukosis virus p15 interacts with interferon regulatory factor 7 to antagonize the cGAS-STING signaling pathway and promote viral replication

**DOI:** 10.1128/jvi.00380-25

**Published:** 2025-07-24

**Authors:** Zhouhao Cui, Kui Shen, Moru Xu, Yongxiu Yao, Hongxia Shao, Aijian Qin, Kun Qian

**Affiliations:** 1 Ministry of Education Key Lab for Avian Preventive Medicine, College of Veterinary Medicine, Yangzhou University 38043, Yangzhou, Jiangsu, China; 2 Jiangsu Key Lab of Preventive Veterinary Medicine, College of Veterinary Medicine, Yangzhou University, Yangzhou, Jiangsu, China; 3 Jiangsu Co-innovation Center for Prevention and Control of Important Animal Infectious Diseases and Zoonoses, Yangzhou University 38043, Yangzhou, China; 4 The Pirbright Institute & UK-China Centre of Excellence for Research on Avian Diseases 111636, Pirbright, United Kingdom; The Ohio State University, Columbus, Ohio, USA

**Keywords:** ALV-J, p15, cGAS-STING signaling pathway, IRF7, immune evasion, viral replication

## Abstract

Cyclic GMP-AMP synthase (cGAS) recognizes viral DNA within the cytoplasm and initiates innate antiviral response through the cGAS-STING pathway. However, viruses have evolved diverse strategies to counteract the cGAS-STING signaling pathway. Avian leukosis virus subgroup J (ALV-J), an avian oncogenic retrovirus, can antagonize host innate immune responses and cause immunosuppression to develop tumors. In this study, with a functional screen, we identified the p15 protein, a protease encoded by ALV-J, inhibiting the cGAS-STING signaling pathway and IFN-β production to promote virus replication. This inhibitory effect is associated with the enzymatic active site of p15. Further study demonstrated that the p15 protein interacted with the DBD domain of interferon regulatory factor 7 (IRF7), inhibiting the dimerization and nuclear translocation of IRF7 but not the phosphorylation of IRF7, resulting in the suppression of IFN-β production. Consistent with these results, small-interfering RNA targeting p15 in ALV-J infection induced more IFN-β in DF-1 cells compared with control RNA. Taken together, these results verified that ALV-J-encoded p15 protein plays a key role in evading the cGAS-STING pathway, which improves our understanding of the virus-host interaction in ALV-J evading host innate immunity and may contribute to developing novel drugs against ALV-J infection.

**IMPORTANCE:**

Among all subgroups, avian leukosis virus subgroup J is one of the most pathogenic, capable of inducing severe malignant tumors and immunosuppressive effects on the infected host. Although there have been some reports on the immunosuppressive mechanisms of ALV-J, the immune evasion mechanism mediated by ALV-J-encoded proteins remains largely unknown. In this study, we found that p15 interacted with IRF7 and inhibited the dimerization and nuclear translocation of IRF7, resulting in the suppression of the expression of IFN-β. Of note, the results demonstrated that the enzymatic active site of p15 (37D, 38S) plays a crucial role in this process. Our findings revealed that p15 enhanced ALV-J replication by inhibiting the cGAS-STING signaling pathway, which highlights the possibility of p15 as a potential drug target.

## INTRODUCTION

The innate immune response serves as the host’s first line of defense against viral infections. When a virus invades host cells, the innate immune system relies on pattern recognition receptors (PRRs) to detect pathogen-associated molecular patterns (PAMPs) on the virus, thereby triggering the production of type I interferons (IFN-I) (1–3). Major PRRs include Toll-like receptors (TLRs), RIG-I-like receptors (RLRs), and NOD-like receptors (NLRs) (4–8). Besides these RNA sensors, several DNA sensors such as interferon gamma-inducible protein 16, DNA-dependent activator of IFN regulatory factors, DEAD box helicase 41, RNA polymerase III, and cyclic GMP-AMP synthase (cGAS) have been identified and characterized (9–13). Among these, cGAS is an important cytoplasmic sensor that recognizes DNA ligands and activates the stimulator of interferon genes (STING) in different cells (11, 14–16).

The cGAS-STING pathway plays a crucial role in the antiviral immune response by stimulating the production of IFN-β. To establish infection, viruses employ various strategies to inhibit the cGAS-STING pathway, thus evading the host’s innate immunity. For example, HSV-1, virus-encoded VP22 interacts with cGAS to inhibit its enzymatic activity (17). VP24 encoded by HSV-1 suppresses IFN-β production by inhibiting the activation of IRF3 (18). In addition, the retrovirus HIV-1 also accomplishes immune evasion by inhibiting the cGAS pathway. It was found that NLRX1 blocks the activation of STING by binding to the STING protein, thereby promoting the replication of HIV-1 in the host (19). Moreover, the HIV-1 viral capsid enables cytoplasmic viral cDNA to evade recognition by cGAS, allowing the virus to continuously replicate without being cleared by the innate immune system (20). In veterinary research, the UL13 of pseudorabies virus (PRV) has been shown to recruit RNF5 to induce STING degradation, thereby inhibiting the cGAS-STING-mediated antiviral response (21). The VP23 protein of Marek’s disease virus (MDV) suppresses the cGAS-STING pathway by inhibiting the activation of IRF7 (22). The E120R protein of the African Swine Fever Virus (ASFV) interacts with IRF3, resulting in the inhibition of its phosphorylation (23). Additionally, the p17 protein of ASFV and Meq protein of Marek’s disease virus (MDV) directly interact with STING, thereby inhibiting its recruitment of TBK1 (24, 25).

Several previous studies showed that ALV-J suppressed the production of IFN by inhibiting the phosphorylation of IκBα (26) and the expression of MDA5 (27). Additionally, ALV-J upregulated the expression of SOCS3 to inhibit the phosphorylation of JAK2/STAT3, resulting in suppression of the host’s innate immune response (28). However, it is not known whether ALV-J can affect the cGAS-STING signaling pathway. In the present study, we demonstrate that ALV-J protein p15 antagonizes the cGAS-STING signaling pathway by interacting with IRF7, inhibiting the IRF7 dimerization and nuclear translocation through its enzymatic active site. Overall, our findings provide new insights into the mechanism of ALV-J immunosuppression, highlighting the development of potential drug targets for the prevention and control of ALV-J infection.

## RESULTS

### ALV-J infection inhibits the activation of the cGAS-STING signaling pathway in DF-1 cells

**Fig 1:**
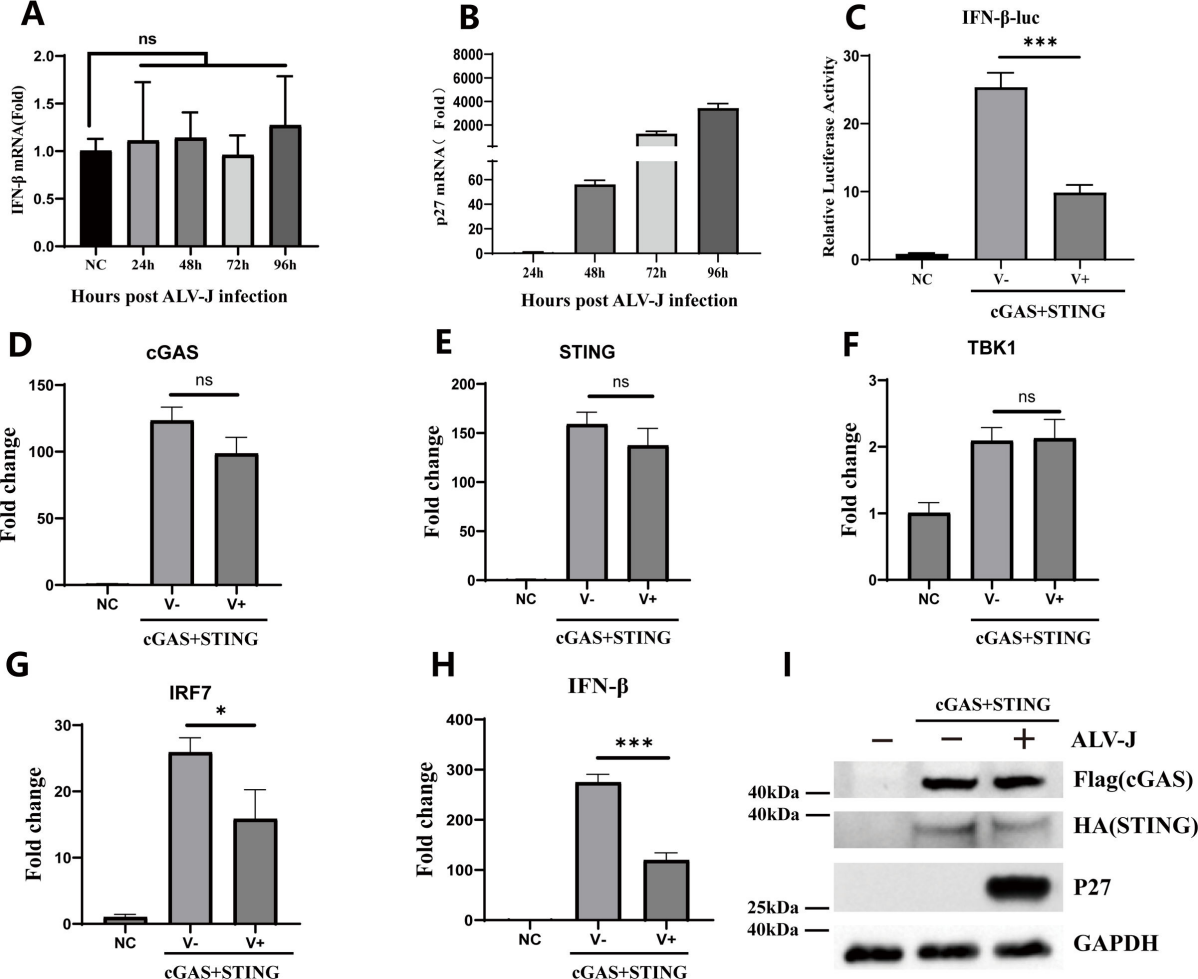
ALV-J infection inhibits the activation of the cGAS-STING signaling pathway in DF-1 cells. (A, B) DF-1 cells were co-transfected with the internal control Renilla luciferase reporter plasmid (pRL-TK) and IFN-β-luc reporter plasmid, followed by infection with JS09GY3 strain at MOI = 1.0. Cells were collected at 24 h, 48 h, 72 h, and 96 h post-infection, and IFN-β mRNA expression levels were assessed by RT-qPCR. (C) DF-1 cells were infected with the JS09GY3 strain at MOI = 1.0, and 24 h post-infection, co-transfected with pRL-TK, chicken cGAS, chicken STING, and IFN-β-luc. DF-1 cells were infected with the JS09GY3 strain at MOI = 1.0, and 24 h post-infection, co-transfected with cGAS and STING, the cGAS (D), STING (E), TBK1 (F), IRF7 (G), and IFN-β (H) mRNA expression levels were assessed using RT-qPCR, and related protein expression levels (I) were analyzed using western blot. Analysis was performed using dual-luciferase reporter assays 24 h post-transfection. Data from three independent experiments are presented as mean ± standard deviation. Statistical significance of differences was determined using the Student *t* test, *P < 0.05.

IFN-β plays a pivotal role in antiviral defense and regulation of the immune response. ALV-J is an oncovirus capable of inducing immune suppression. Further elucidation is required to understand how ALV-J regulates the production of IFN-β. Here, we infected the cells with ALV-J and observed no significant difference in the expression levels of IFN-β during virus replication at different time points in DF-1 cells (Fig. 1A and B). Then, we established a dual-luciferase reporter system by co-transfecting cells with an IFN-β-luciferase reporter plasmid and a reference internal Renilla luciferase reporter plasmid (pRL-TK). When co-transfected with cGAS and STING, the IFN-β promoter activity was markedly downregulated in the virus-infected group (Fig. 1C). The results confirmed that ALV-J virus infection does not affect the gene expression levels of cGAS, STING, and TBK1 but significantly inhibits the gene expression levels of IRF7 and IFN-β induced by cGAS-STING (Fig. 1D through H). In addition, ALV-J infection mildly downregulated the protein levels of STING but did not affect the protein levels of cGAS (Fig. 1I). These findings suggest that viral-encoded proteins have an inhibitory effect on the activation of the cGAS-STING-signaling pathway during viral infection.

### The enzymatic activity sites of p15 are crucial for its inhibitory effect on the cGAS-STING pathway

**Fig 2:**
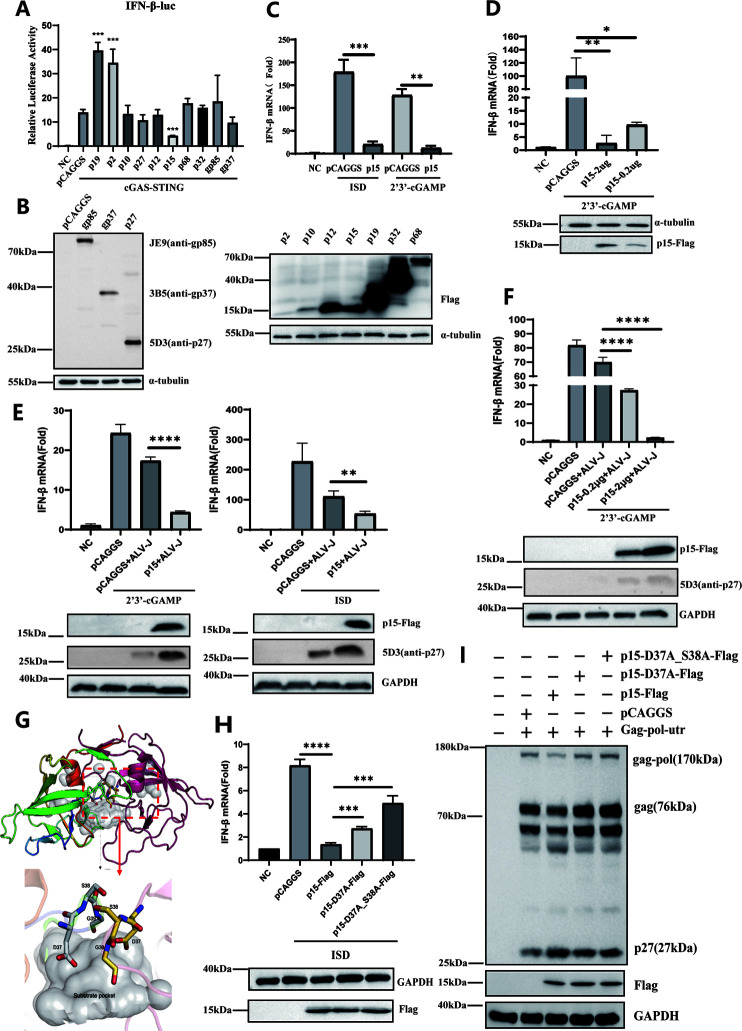
p15 inhibits the production of IFN-β mediated by the cGAS-STING pathway associated with its enzymatic activity. Co-transfection of chicken pCAGGS-cGAS-Flag, chicken pCAGGS-STING-HA, reporter plasmids pGL3-IFN-β, pRL-TK, and one of the viral protein expression plasmids. Dual-luciferase reporter assays were performed 36 h post-transfection (A); the expression levels of 10 proteins encoded by the virus were detected using western blot (B). (C, D) Transfection of empty vector or p15 expression plasmid into DF-1 cells, followed by stimulation with pathway-specific activators ISD and 2’3’-cGAMP for 24 h, respectively. IFN-β gene expression levels were assessed using RT-qPCR, and p15 protein expression was analyzed using western blot. (E, F) DF-1 cells were transfected with either an empty vector or a p15 expression plasmid, infected with ALV-J virus, and then stimulated with the pathway-specific activators ISD and 2’3’-cGAMP for 24 h, respectively. IFN-β gene expression levels were assessed by RT-qPCR, and p15 protein expression was analyzed using western blot. (G) The enzymatic activity site of the p15 protein was predicted using the software Swiss-Model. (H) Empty vector, p15-Flag, p15-D37A-Flag, and p15-D37A_S38A-Flag were transfected into DF-1 cells, followed by transfection with ISD (1 µg per well) 24 h later. IFN-β gene expression levels were measured by RT-qPCR, and p15, P15-D/A, and P15-D/A_S/A protein expression levels were assessed by Western Blot. (I) Empty vector, p15, p15-D37/A, p15-D37/A_S38/A were co-transfected with Gag-pol-utr into DF-1 cells. The P27 protein was assessed using western blot. Data from three independent experiments are presented as mean ± standard deviation. Statistical significance of differences was determined by Student *t* test (*P < 0.05; **P < 0.01; ***P < 0.001; ****P < 0.0001).

To screen for viral-encoded proteins that inhibit the cGAS-STING pathway, we used an IFN-β dual-luciferase reporter system with co-transfection of cGAS and STING. Among the 10 ALV-J-encoded proteins, the p15 protein significantly downregulated IFN-β promoter activity (Fig. 2A). To further confirm the inhibitory effect of p15 on the cGAS-STING pathway, two specific activators of the pathway, ISD (1 µg) and 2’3’-cGAMP (1 µg/mL), were used in DF-1 cells. The results further confirmed that p15 inhibits the production of IFN-β induced by ISD and 2’3’-cGAMP (Fig. 2C) in a dose-dependent manner (Fig. 2D). Similar results were also obtained in the case of ALV-J virus infection (Fig. 2E and F).

As we know, p15 is an essential protease during ALV-J replication, which cleaves precursor proteins encoded by the gag and pol genes (29). Its enzymatic active site was predicted using the Swiss Model (Fig. 2G). To investigate whether p15 inhibition of the cGAS-STING pathway is dependent on its enzymatic activity, we constructed two plasmids with single and double mutations in the enzymatic active site of p15 (p15-D37A-Flag and p15-D37A_S38A-Flag). Subsequently, we transfected empty vector, p15, and enzymatic active site mutant plasmids into DF-1 cells and stimulated them with ISD. The results showed that p15 significantly downregulated the gene expression level of IFN-β compared to the control group, whereas the inhibitory effect of p15 was significantly recovered after mutations in the p15 protein (Fig. 2H). To further validate the impact of point mutations on the activity of p15, we co-transfected p15 and its mutants’ plasmids with Gag-pol-utr. Monoclonal antibody 5D3 can recognize gag pol protein (170 kDa), gag protein (76 kDa), p27 protein (27 kDa), and other intermediate products during protein processing (30). Compared with the empty vector control, the levels of mature p27 were significantly upregulated after transfection with the wild-type p15, indicating that the gag protein encoded by the virus was cleaved by the p15 protein. However, after the active site of p15 was mutated, it could not induce the production of a more mature p27 protein (Fig. 2I). These results indicate that amino acids at positions 37 and 38 of p15 constitute its active site, which plays a crucial role in the inhibition of the cGAS-STING pathway.

### p15 inhibits the cGAS-STING pathway by targeting IRF7

**Fig 3:**
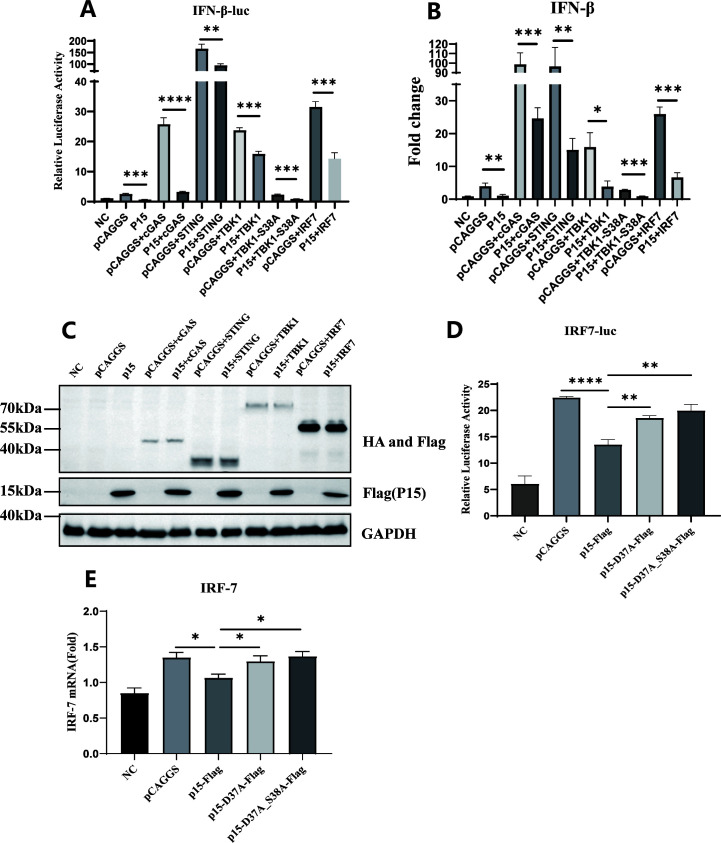
p15 inhibits IFN-β production by targeting IRF7. Empty vector or p15 expression plasmids were co-transfected with cGAS, STING, TBK1, TBK1-S38A, and IRF7 into DF-1 cells, respectively. 48 h post-transfection, IFN-β promoter activity was analyzed using dual-luciferase reporter assays (A), and gene expression levels of IFN-β were measured by RT-qPCR (B), and the related protein expression levels (C) were analyzed using western blot. Empty vector, p15, p15-D37A-Flag, and p15-D37A_S38A-Flag were transfected into DF-1 cells, and 24 h later, 2’3’-cGAMP (1 µg/mL) was added. The promoter activity of IRF7 was assessed using dual-luciferase reporter assays (D). Gene expression levels of IRF7 were measured by RT-qPCR (E). Data from three independent experiments are presented as mean ± standard deviation. Statistical significance of differences was determined by Student *t*-test (*P < 0.05; **P < 0.01; ***P < 0.001; ****P < 0.0001).

In order to clarify the target of p15-induced inhibition of the cGAS-STING pathway, we co-transfected empty vector or p15 expression plasmids with cGAS, STING, TBK1, and IRF7 into DF-1 cells, along with IFN-β-luciferase reporter and pRL-TK. The results demonstrated that p15 does not affect the protein expression co-transfected with it but significantly inhibits the promoter activity and mRNA level of IFN-β induced by cGAS, STING, TBK1, and IRF7 (Fig. 3A through C), suggesting that p15 inhibited the cGAS-STING signaling pathway by targeting IRF7 or downstream. Subsequently, we transfected empty vector, p15, p15-D37A-Flag, and p15-D37A_S38A-Flag into DF-1 cells, followed by performing dual-luciferase assays and RT-qPCR. The results revealed that p15 significantly downregulated the promoter activity and gene expression level of IRF7 under 2’3’-cGAMP stimulation, and this effect was completely restored by enzyme site mutated p15 protein (Fig. 3D and E). Unfortunately, due to the lack of commercial IRF7 antibodies for poultry, we are unable to detect the endogenous IRF7 protein. These results suggest that p15 inhibits the production of IFN-β from the activation of the cGAS-STING pathway by targeting IRF7 in DF-1 cells.

### p15 inhibits the dimerization and nuclear translocation of IRF7, but not its phosphorylation

**Fig 4:**
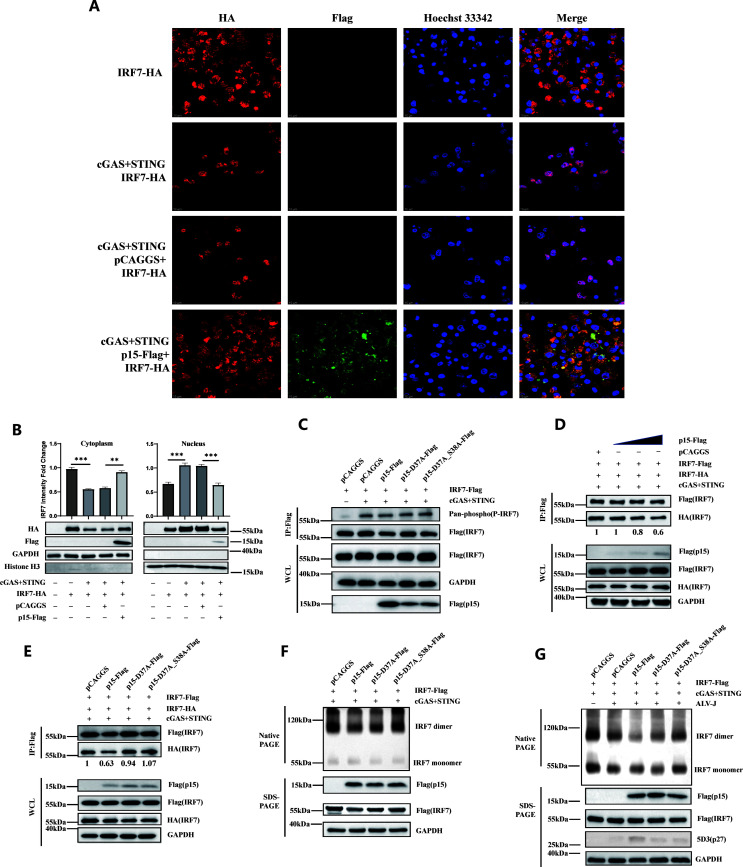
p15 inhibits IRF7 dimerization and nuclear translocation. Empty vector or p15 was co-transfected with IRF7 into HD11 cells. After 24 h, chicken cGAS and chicken STING were transfected. IRF7 nuclear translocation was observed using confocal microscopy (A), and the expression of IRF7 in the cytoplasm and nucleus was detected using western blot (B). (C) Empty vector, p15, p15-D37/A, and p15-D37/A_S38/A were co-transfected with IRF7 into DF-1 cells. After 36 h, chicken cGAS and chicken STING were transfected, and IRF7-Flag was immunoprecipitated using Flag-beads. Phosphorylation levels of IRF7 were detected using western blot using a phospho-specific antibody. We co-transfected IRF7-HA and IRF7-Flag, and through immunoprecipitation of IRF7-Flag, detected IRF7-HA to assess the level of IRF7 dimerization. (D) Empty vector or p15-Flag was co-transfected with IRF7-Flag and IRF7-HA into DF-1 cells. After 36 h, chicken cGAS and chicken STING were transfected, and IRF7-Flag was immunoprecipitated using Flag-beads. The binding of IRF7-HA with IRF7-Flag was detected using western blot. (E) Empty vector, p15-Flag, p15-D37/A-Flag, and p15-D37/A_S38/A-Flag were co-transfected with IRF7-Flag and IRF7-HA into DF-1 cells. After 36 h, chicken cGAS and chicken STING were transfected, and IRF7-Flag was immunoprecipitated using Flag-beads. The binding of IRF7-HA with IRF7-Flag was detected by Western blot. Empty vector, p15, p15-D37A, and p15-D37A_S38A were co-transfected with IRF7 into DF-1 cells, with (F) or without (G) ALV-J infection. After 36 h, chicken cGAS and chicken STING were transfected, and IRF7 dimerization was assessed using non-denaturing PAGE. WCL: whole cell lysate.

During the activation of the cGAS-STING signaling pathway, cytoplasmic IRF7 undergoes dimerization and translocation into the nucleus, facilitating the transcription of the IFN-β gene to counteract viral infection (31). Therefore, we first investigated the effects of p15 on IRF7 dimerization and nuclear translocation. After 24 h of co-transfection of IRF7 with either the empty vector or p15, HD11 cells were co-transfected with cGAS and STING. The nuclear translocation of IRF7 was observed using confocal microscopy. The results revealed that the presence of p15 inhibited the nuclear translocation of IRF7 in HD11 cells (Fig. 4A). Furthermore, western blotting analysis confirmed that p15 significantly inhibited the nuclear translocation of IRF7 protein (Fig. 4B).

During the activation of the cGAS-STING signaling pathway, IRF7 undergoes phosphorylation followed by dimerization and nuclear translocation. To further elucidate the effect of p15 on IRF7 phosphorylation and dimerization, we co-transfected empty vector, p15, and its mutant plasmids along with IRF7 into DF-1 cells stimulated by co-transfection of cGAS and STING expression plasmids. The results of co-immunoprecipitation and western blot analysis showed that p15 did not affect the phosphorylation level of IRF7 (Fig. 4C). However, the p15 significantly inhibited IRF7 dimerization in a dose-dependent manner (Fig. 4D). In addition, the inhibitory effect of p15 is confirmed with western blot in native gel (Fig. 4F and G). Importantly, we found that the enzymatic active site of p15 plays a crucial role in inhibiting IRF7 dimerization. The results revealed that p15 ceased to inhibit IRF7 dimerization after mutations occurred in amino acid residues 37D and 38S of p15 (Fig. 4E through G). Overall, these findings suggest that p15 exerts significant inhibition on IRF7 dimerization and nuclear translocation, relying on its enzymatic activity.

### Interaction between p15 and IRF7

**Fig 5:**
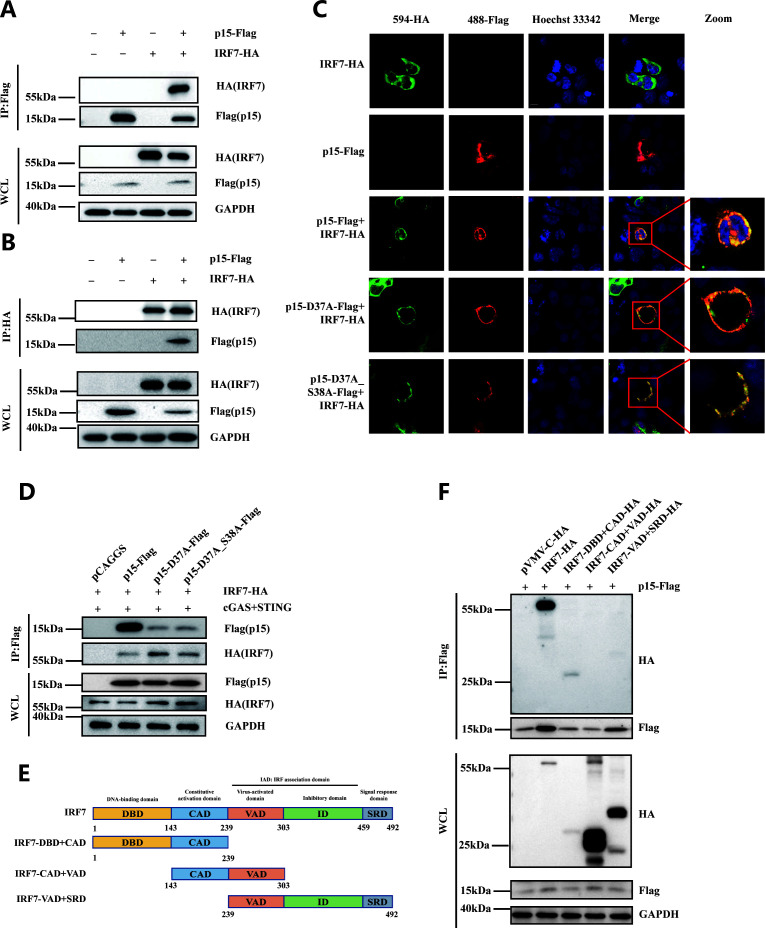
p15 interacts with IRF7. p15-Flag and IRF7-HA were co-transfected into DF-1 cells, and 48 h later, immunoprecipitation was performed using Flag-beads (A) and HA-beads (B) to co-immunoprecipitate p15-Flag and IRF7-HA. (C) p15-Flag and/or IRF7-HA were transfected into 293T cells. After 48 h, cells were fixed, and immunostaining was performed using rabbit anti-Flag and mouse anti-HA as primary antibodies, followed by Alexa Fluor 488-conjugated anti-mouse and Alexa Fluor 594-conjugated anti-rabbit secondary antibodies. Cell nuclei were stained with Hoechst 33342. Co-localization is shown in yellow in the merged images. (D) Empty vector, p15-Flag, p15-D37/A-Flag, and p15-D37/A_S38/A-Flag were co-transfected with IRF7-HA into DF-1 cells. After 36 h, chicken cGAS and chicken STING were transfected, and p15-Flag was immunoprecipitated using Flag-beads. IRF7-HA was detected using western blot. (E) Full-length IRF7 (aa 1-492) and various truncated forms of IRF7, including IRF7-DBD + CAD (aa 1-239), IRF7-CAD + VAD (aa 143-303), and IRF7-VAD + SRD (aa 239-492) are shown. (F) p15-Flag was co-transfected with full-length IRF7-HA or truncated forms of IRF7-HA into DF-1 cells. After 48 h, immunoprecipitation was performed using Flag-beads, and western blot analysis was conducted. WCL: whole cell lysate.

The specific inhibition of IRF7 by p15 suggests a possible interaction between the two proteins. To verify this hypothesis, the p15-Flag and IRF7-HA were co-transfected into DF-1 cells, and performed immunoprecipitation experiments to detect their interaction. We found that IRF7 can be immunoprecipitated with p15 (Fig. 5A); conversely, p15 can also be immunoprecipitated with IRF7 (Fig. 5B). Co-localization experiments further confirmed the interaction between p15 and IRF7. Of note, mutations in amino acid residues 37D and 38S of p15 did not affect their interaction (Fig. 5C and D). These results indicate that there is indeed an interaction between the p15 and IRF7 proteins.

To further elucidate the interaction domains of IRF7 with p15, we constructed a series of truncated mutants of IRF7 (Fig. 5E). As depicted in Fig. 5F, IRF7 (1-492) and IRF7-DBD + CAD (1-239) constructs were able to interact with p15, whereas IRF7-CAD + VAD (143-303) and IRF7-VAD + SRD (239-492) were not co-immunoprecipitated with p15. These results suggest that the DBD domain of IRF7 is crucial for its interaction with p15.

### p15 inhibits the production of IFN-β mediated by the cGAS-STING pathway and promotes viral replication

**Fig 6:**
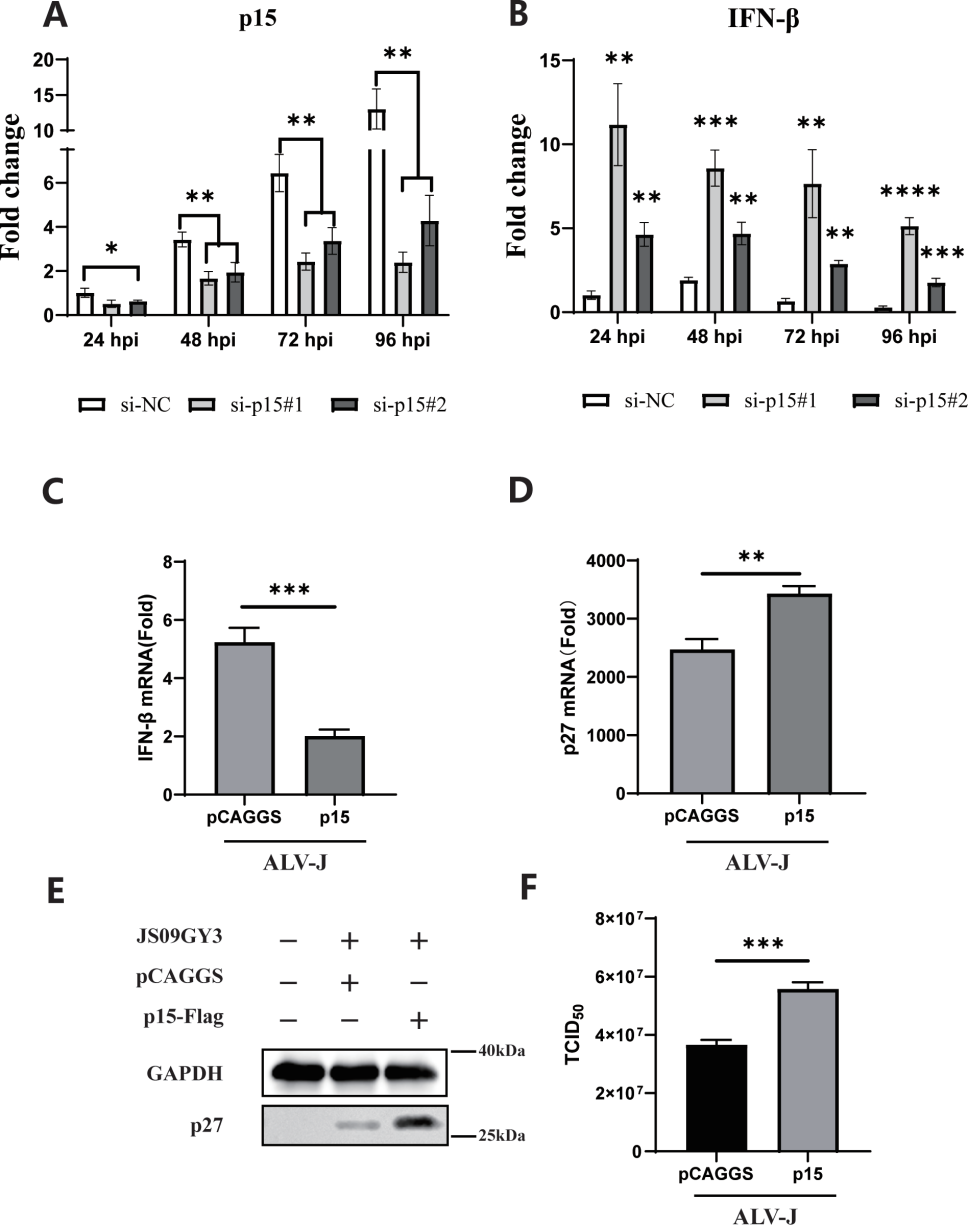
p15 inhibits the production of IFN-β mediated by the cGAS-STING pathway and promotes viral replication. DF-1 cells were infected with the JS09GY3 strain at an MOI of 1.0, followed by co-transfection with interference RNA targeting p15 with chicken cGAS and chicken STING. The total RNA was extracted from the infected cells collected at 24 h, 48 h, 72 h, and 96 h post-infection. RT-qPCR was performed to detect the gene expression levels of p15 (A) and IFN-β (B). DF-1 cells were transfected with pCAGGS empty vector or pCAGGS-p15-Flag, followed by infection with JS09GY3 strain at MOI = 1.0. Cells were harvested at 96 h post-infection. Gene expression levels of IFN-β (C) and P27 (D) were assessed by RT-qPCR, and p27 protein levels were detected using western blot (E). The virus titers in the cell culture supernatant were determined (F). Data from three independent experiments are presented as mean ± standard deviation. Statistical significance of differences was determined by Student *t*-test (*P < 0.05; **P < 0.01; ***P < 0.001; ****P < 0.0001).

We initially intended to construct ALV-J strains with mutations in the enzymatic active site of p15. However, subsequent studies revealed that mutations in the enzymatic active site of p15 (37D, 38S) are lethal for the virus (unpublished data). Therefore, we synthesized small interfering RNA targeting p15 to examine the repercussions of inhibiting p15 expression on viral replication. The results revealed that during ALV-J virus infection, knocking down p15 expression significantly enhanced the production of IFN-β mediated by cGAS-STING transfection compared with the control interference RNA (Fig. 6B).

To further validate the role of p15 in viral replication, we transfected empty vector or p15 expression plasmids into DF-1 cells and subsequently infected them with the JS09GY3 strain of ALV-J at MOI = 1.0. The results showed that overexpression of p15 significantly downregulated the gene expression level of IFN-β (Fig. 6C), upregulated the gene (Fig. 6D) and protein (Fig. 6E) expression levels of the viral P27 protein, and increased the viral titer in the cell culture supernatant also (Fig. 6F). These results indicate that overexpression of p15 significantly promotes viral replication.

## DISCUSSION

Avian leukosis virus (ALV) represents a significant immunosuppressive threat in poultry. ALV-J infection in chicken flocks renders them more vulnerable to secondary viral and bacterial infections, impairing the efficacy of vaccinations and resulting in immune failure and substantial economic losses (32). Although several studies have delved into the immunosuppressive mechanisms of ALV-J, how the ALV-J encoded proteins contribute to these immune suppressions and the detailed mechanisms remain unknown.

Since the discovery of the cGAS-STING pathway, cGAS has emerged as the primary cytoplasmic DNA sensor, playing a pivotal role in responding to various viral infections (11). It activates downstream signaling molecules, namely STING, to induce the production of IFN-β and exert its antiviral function. Widely recognized as a critical pathway in innate immunity (33), the cGAS-STING signaling pathway has been extensively studied in the recognition of DNA and RNA viruses. Although retroviruses have received less attention in this context, there is evidence linking viruses, such as HIV, to the antiviral innate immune response (34). In this study, we discovered that ALV-J, an avian retrovirus, suppressed the activation of the cGAS-STING pathway in DF-1 cells (Fig. 1). Subsequently, with a functional screen, we found that only the p15 protein significantly inhibited the expression of IFN-β mediated by the cGAS-STING pathway (Fig. 2A and C through F).

The ALV-J p15 protein, encoded by the gag gene, plays a crucial role as both a viral nucleocapsid protein and a protease. It cleaves precursor proteins encoded by the gag and pol genes to generate mature proteins and peptides (35). Additionally, the N-terminal region, comprising the first seven amino acids, is implicated in viral assembly (36). In the present study, we also observed that p15 promoted ALV-J replication (Fig. 6C through F). Through bioinformatics analysis, we identified two critical enzymatic active sites of p15, which were located at amino acids 37D and 38S (Fig. 2G). In Fig. 2H, the enzyme activity mutants were still able to slightly inhibit IFN-β, suggesting that the inhibition of IFNβ production by P15 is associated with its enzymatic active site. However, there are some other unknown factors at play. It needs further study to address it. Upon analysis and comparison, we observed a high degree of conservation in the p15 sequence among different subgroups of ALV (data not shown). Particularly noteworthy is the observation that p15 proteins from different subgroups possess identical enzymatic active sites, implying that all p15 proteins encoded by other subgroups may have similar functions and mechanisms.

To elucidate the mechanism by which p15 inhibits cGAS-STING pathway-mediated IFN-β production, we identified IRF7 as the target of p15 in the cGAS-STING signaling pathway. IRF7 serves as an essential nuclear regulatory factor in the cGAS-STING pathway. During the antiviral response, IRF7 undergoes phosphorylation and dimerization, ultimately translocating into the nucleus to facilitate the production of IFN-β (37). Several viruses target IRF7 to escape innate immunity, such as the σ A protein of avian reovirus (38), the VP23 protein of Marek’s disease virus (22), the US3 protein of Marek’s disease virus (39), and the US3 protein of duck hepatitis virus (40). In the present investigation, we discovered that the ALV-J p15 protein interacted with IRF7 (Fig. 5A through C), consequently inhibiting the dimerization and nuclear translocation of IRF7, but not phosphorylation (Fig. 4A through G), leading to the decrease of IFN-β expression. In this process, the enzymatic activity site of p15 plays a crucial role. After the mutations at positions 37D and 38S of p15, although it does not affect its interaction with IRF7 (Fig. 5C), it significantly weakens its ability to inhibit IRF7 dimerization (Fig. 4E). Additionally, this study found that p15 specifically interacts with the DBD domain of IRF7 (1–143 aa) (Fig. 5F). The DBD domain of IRF7 plays a crucial role in binding to the interferon-stimulated response element. According to the previous report, the P200 family IFI204 protein can specifically interact with the DBD domain of IRF7, thereby inhibiting its subsequent binding to promoter DNA (41). Hence, we speculate that p15 might inhibit the binding of IRF7 to promoter DNA by interacting with its DBD domain. The further mechanism needs more studies to clarify.

**Fig 7:**
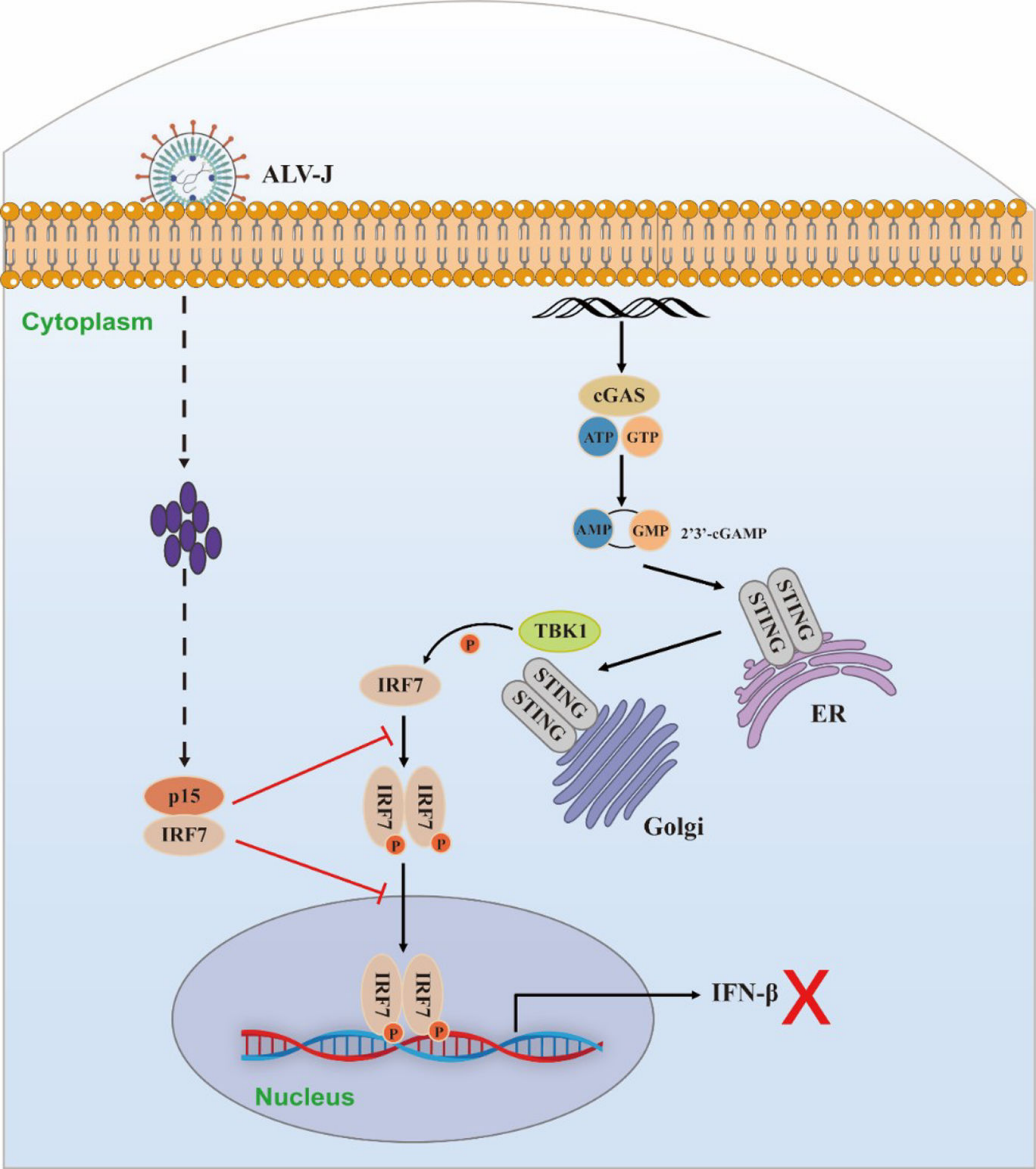
Schematic representation of p15 inhibiting the cGAS-STING pathway. Under normal circumstances, the activation of the cGAS-STING pathway triggers the recruitment of TBK1, subsequently leading to the phosphorylation and dimerization of IRF7. The activated IRF7 translocates into the nucleus, where it initiates the production of IFN-β. However, during ALV-J virus infection, the virus-encoded p15 protein interacts with IRF7, disrupting the formation of IRF7 dimers and preventing their nuclear translocation. Consequently, this inhibition results in the suppression of IFN-β production.

In summary, this study unveils that the p15 protein encoded by ALV-J utilizes its enzymatic activity to impede cGAS-STING-induced IFNβ production and promote viral replication. The specific inhibitory mechanism entails p15 interacting with IRF7, hampering its dimerization and nuclear translocation, thereby suppressing IFNβ production (Fig. 7). These findings reveal a novel mechanism by which ALV-J antagonizes the cGAS-STING signaling pathway, providing novel insights and clues for the prevention and control of avian leukosis virus.

## MATERIALS AND METHODS

### Virus, cells, and antibodies

The ALV-J JS09GY3 strain (GenBank accession number GU982308.1) was maintained by the Key Laboratory of Preventive Veterinary Medicine for Poultry of the Ministry of Education at Yangzhou University. DF-1, HD11, and HEK293T cells were cultured at 37°C with 5% CO2_2_. Commercially available antibodies included mouse anti-Flag, mouse anti-HA (Engibody, USA), rabbit anti-HA (CST, USA), mouse anti-GAPDH, mouse anti-pan Phospho-Serine/Threonine (ABclonal, China), mouse anti-α-tubulin (Sigma-Aldrich, USA), rabbit anti-Flag, mouse anti-histone H3 (Beyotime, China), and 5D3 (anti-p27), JE9 (anti-gp85), and 3B5 (anti-gp37) were prepared in our laboratory.

### Plasmid construction

Expression plasmids for ALV-J-encoded proteins, pcDNA3.1-p27, pCAGGS-p2-Flag, pCAGGS-p10-Flag, pCAGGS-p12-Flag, pCAGGS-p15-Flag, pCAGGS-p19-Flag, pCAGGS-p32-Flag, pCAGGS-p68-Flag, pCAGGS-gp85-Flag, pCAGGS-gp37-Flag, IRF7 expression plasmid pcDNA3.1-IRF7-IgG, cGAS expression plasmid pCAGGS-cGAS, STING expression plasmid pCAGGS-STING, Gag-pol-utr (30), as well as p15 single mutation plasmid pCAGGS-p15-D37A-Flag and double mutation plasmid pCAGGS-p15-D37A_S38A-Flag were constructed in our laboratory. Primers were designed to amplify the IRF7 gene using pcDNA3.1-IRF7-IgG as a template and clone into pcDNA3.1, with HA or Flag tags at the 3' end, generating pcDNA3.1-IRF7-HA and pcDNA3.1-IRF7-Flag constructs. The truncated IRF7s, including IRF7-DBD + CAD (aa 1–239), IRF7-CAD + VAD (aa 143–303), and IRF7-VAD + SRD (aa 239–492), were amplified using pcDNA3.1-IRF7-HA as a template, and the corresponding fragments were cloned into pCMV-C-HA. The primers are presented in Table 1.

**TABLE 1:** Primers used in this research

Primer name	Sequence (5’—3’)	Purpose
IRF7-HA-F	CGGGGTACCATGGCAGCACTGGACAGCG	Gene cloning
IRF7-HA-R	CCGGAATTCTCAAGCGTAGTCTGGGACGTCGTATGGGTAGTCTGTCTG CATGTGGTATTGCTC	Gene cloning
IRF7-Flag-F	CGGGGTACCATGGCAGCACTGGACAGCG	Gene cloning
IRF7-Flag-R	CCGGAATTCTCACTTATCGTCGTCATCCTTGTAATCGTCTGTCTG CATGTGGTATTGCTC	Gene cloning
IRF7-DBD + CAD F	GGAATTCATGGCAGCACTGGACAGCG	Gene cloning
IRF7-DBD + CAD R	GGGTCGACACAGCCCCCACCTGTGAG	Gene cloning
IRF7-CAD + VAD F	GGAATTCATGGATTTGGCCTTGGAAAACACTCC	Gene cloning
IRF7-CAD + VAD R	GGGTCGACGTCCACCTGCTCCTGGTAGACCA	Gene cloning
IRF7-VAD + SRD F	GGAATTCATGGGGCAGGACGGGGCT	Gene cloning
IRF7-VAD + SRD R	GGGTCGACGTCTGTCTGCATGTGGTATTGCTC	Gene cloning
qIFN-β-F	GCCCACACACTCCAAAACACTG	qRT-PCR
qIFN-β-R	TTGATGCTGAGGTGAGCGTTG	qRT-PCR
qIRF7-F	GAGCCTCCTCCCTCAACAGT	qRT-PCR
qIRF7-R	AGGGACACAGGAAGGGAGTG	qRT-PCR
q18S-F	TCAGATACCGTCGTAGTTCC	qRT-PCR
q18S-R	TTCCGTCAATTCCTTTAAGTT	qRT-PCR
p27-F	CCGGGGAATTGGTTGCTAT	qRT-PCR
p27-R	AGTCAATGATCACCGGAGCC	qRT-PCR
p15-F	GTCATCCAGTCAAACAGCGT	qRT-PCR
p15-R	GCCAATCAGTAGGCCAATCC	qRT-PCR

### Real-time qPCR (qPCR)

Total RNA was extracted using the FastPure Cell/Tissue Total RNA Isolation Kit V2 (Vazyme, China), and reverse transcription into cDNA was performed using the HiScript III RT SuperMix for qPCR (Vazyme, China). ChamQ Universal SYBR qPCR Master Mix (Vazyme, China) was used for quantitative PCR to detect the gene expression level of P27, IFNβ, cGAS, STING, TBK1, NF-κB, and IRF7, with 18S as the reference gene. All samples were run in triplicate. The primers are presented in Table 1.

### Transfection and dual-luciferase assay

The Lipofectamine 3000 transfection reagent (Invitrogen, China) was used to co-transfect the firefly luciferase reporter (40 ng) (IFN-β-luc or IRF7-luc) and the Renilla luciferase reporter (10 ng) (pRL-TK) into DF-1 cells as an internal control. Simultaneously, the expression plasmids were co-transfected as experimental groups. Cells were collected 36 h post-transfection, and the dual-luciferase assay kit was employed to measure firefly and Renilla luciferase activities. All samples were run in triplicate.

### Immunoprecipitation (IP) and western blotting

Plasmids were transfected into HEK293T or DF-1 cells using Lipofectamine 3000 transfection reagent (Invitrogen, China). After 48 h of transfection, cells were lysed with western and IP lysis buffer (NCM, China) containing protease inhibitors (NCM, China). Anti-Flag M2 affinity gel (Sigma-Aldrich, USA) or anti-HA affinity gel (Beyotime, China) was added to the collected cell samples and incubated overnight at 4°C. The affinity gel was washed five times with pre-chilled phosphate-buffered saline (PBS), boiled in 1 × SDS-loading buffer for 5 min, and then subjected to western blotting to detect IP proteins.

For western blotting experiments, total cellular proteins were extracted using RIPA lysis buffer (NCM, China) with protease and phosphatase inhibitors (NCM, China). Cytoplasmic and nuclear proteins were extracted using a Cell Nuclear Protein and Cytoplasmic Protein Extraction Kit (Beyotime, China). Protein concentration was determined using the BCA Protein Quantification Kit (Vazyme, China). The separated proteins by SDS-PAGE were transferred to NC membranes, blocked with 5% skimmed milk, incubated with primary and secondary antibodies, and finally detected using an ECL chemiluminescence system (Tanon, China) to visualize the target proteins.

### Confocal immunofluorescence experiment

Plasmids were transfected into HD11 or HEK293T cells, and 48 h post-transfection, the cells were fixed with pre-chilled 4% paraformaldehyde for 20 min. Permeabilization was done with 0.25% Triton X-100 for 10 min, followed by blocking with 2% goat serum for 1 h. The cells were then incubated with mouse anti-IRF7 or mouse anti-Flag or/and rabbit anti-HA antibodies for 1 h. Afterward, the cells were incubated with secondary antibodies Alexa Fluor 488 goat anti-mouse and Alexa Fluor 594 goat anti-rabbit (Jackson, USA) for 1 h. Finally, cell nuclei were stained with Hoechst 33342 (Jackson, USA) for 8 min. Images were captured using a Zeiss LSM880 confocal microscope (Zeiss, Germany).

### Non-denaturing PAGE

Cells were lysed using western and IP lysis buffer (NCM, China) containing protease inhibitors (NCM, China). The protein samples were mixed with 5 × non-denaturing non-reducing buffer (Beyotime, China) and subjected to electrophoresis using a Tris-glycine buffer system (without SDS) under a constant voltage of 80V until the samples entered the concentrating gel, followed by a change in voltage to 100V. An anti-Flag monoclonal antibody was used to detect IRF7-Flag monomers and dimers.

### ALV-J virus infection and TCID50_50_ determination

DF-1 cells were infected with ALV-J at a multiplicity of infection (MOI) of 1. At 2 h post-infection, the medium was replaced with a maintenance medium. Cell culture supernatant was collected for TCID50_50_ determination. Briefly, the collected cell culture supernatant was serially diluted in the medium (10-1^−1^, 10-2^−2^, 10-3^−3^, ..., 10-11^−11^), and used to infect DF-1 cells in a 96-well plate. After 7 days of incubation, immunofluorescence was performed for detection. The primary antibody used was a laboratory-made p27 monoclonal antibody, and the secondary antibody was FITC-labeled goat anti-mouse. TCID50_50_ was calculated after observation under a fluorescence microscope.

### RNA interference assay

Three siRNAs targeting p15 of ALV-J were designed and synthesized by Suzhou Jima Pharmaceutical Technology; 150 pmol of siRNAs or the negative control siRNA (siNC) was transfected into DF-1 cells infected with ALV-J (MOI = 1) using Lipofectamine reagents (Invitrogen, Shanghai, China). Total RNA was extracted for RT-qPCR at 24 h, 48 h, 72 h, and 96 h postinfection (hpi). The siRNA sequences are presented in Table 2.

**TABLE 2:** siRNA sequences

siRNA primer name	Sequence (5’—3’)
si-NC (sense)	UUCUCCGAACGUGUCACGUTT
si-NC (antisense)	ACGUGACACGUUCGGAGAATT
si-p15 (sense)	GCGAUGACAAUGGAACAUATT
si-p15 (antisense)	UAUGUUCCAUUGUCAUCGCTT

### Data analysis

The data are presented as mean ± standard deviation (SD). Significance analysis of variation between experiments was conducted using GraphPad Prism v10.0 software. Differences in data were assessed using the Student *t*-test.

## Data Availability

The data that support the findings of this study are available on request from corresponding author Kun Qian upon reasonable request.
